# Stride Counting in Human Walking and Walking Distance Estimation Using Insole Sensors

**DOI:** 10.3390/s16060823

**Published:** 2016-06-04

**Authors:** Phuc Huu Truong, Jinwook Lee, Ae-Ran Kwon, Gu-Min Jeong

**Affiliations:** 1 Department of Electrical Engineering, Kookmin University, Seoul 02707, Korea; phtruong@kookmin.ac.kr; 2 3L Labs Co., Ltd., Gasan-dong, 60-4, Geumcheon-gu, Seoul 08512, Korea; jw.lee@3llab.com; 3 College of Herbal Bio-Industry, Daegu Haany University, Gyeongsan 38610, Korea; arkwon@dhu.ac.kr

**Keywords:** gait monitoring, walking distance, insole sensors

## Abstract

This paper proposes a novel method of estimating walking distance based on a precise counting of walking strides using insole sensors. We use an inertial triaxial accelerometer and eight pressure sensors installed in the insole of a shoe to record walkers’ movement data. The data is then transmitted to a smartphone to filter out noise and determine stance and swing phases. Based on phase information, we count the number of strides traveled and estimate the movement distance. To evaluate the accuracy of the proposed method, we created two walking databases on seven healthy participants and tested the proposed method. The first database, which is called the short distance database, consists of collected data from all seven healthy subjects walking on a 16 m distance. The second one, named the long distance database, is constructed from walking data of three healthy subjects who have participated in the short database for an 89 m distance. The experimental results show that the proposed method performs walking distance estimation accurately with the mean error rates of 4.8% and 3.1% for the short and long distance databases, respectively. Moreover, the maximum difference of the swing phase determination with respect to time is 0.08 s and 0.06 s for starting and stopping points of swing phases, respectively. Therefore, the stride counting method provides a highly precise result when subjects walk.

## 1. Introduction

Gait analysis is an important topic in recent research because it provides an effective method for healthcare and medical treatment. The analysis and monitoring of gaits facilitate the development of useful applications in geriatrics [1,2], Parkinson’s disease [3], and rehabilitation [4]. The rapid development of inertial sensor technology has enhanced data collecting methods based on human movement. Thus, significant research has been undertaken to analyze the human gait cycle. Tong *et al.* [5] used uni-axial gyroscopes attached to the leg shank and thigh segment to record angular velocity for each segment. Using these velocities, they derived the segment inclination and knee angle to estimate stride length and gait phases. Pappas *et al.* [6] placed a gyroscope and three force sensitive resistors on the shoe sole to create a gait phase detection system that can detect in real-time four gait phases, *i.e.*, stance, heel-off, swing and heel-strike. Aminian *et al.* [7] attached gyroscope sensors into each shin and on the right thigh to collect angular speed data, and then estimated stride length and velocity based on wavelet analysis.

Recently, an emerging application of gait analysis is the measurement of human walking distance [7,8,9,10]. Walking distance is an important factor to calculate the energy consumption in healthcare applications [11]. Moreover, walking distance estimation is also applied in creating Pedestrian Dead Reckoning (PDR) for indoor localization [12,13]. Hence, considerable research has been conducted to estimate walking distance [9,10,14]. Specifically, Wang *et al.* [9] created the signal magnitude subtraction (SMS) feature of acceleration to detect gait phases. Then, they used a linear regression model to estimate the step velocity from the acceleration data and created second linear regression model to estimate the step length to calculate the walking distance. In [14], Fortune *et al.* proposed an adaptive threshold-based algorithm for step counting and cadence calculation using triaxial acceleration. Yun *et al.* [10] combined acceleration data with orientation estimated from three angular rate sensors and three orthogonal magnetometers to calculate walking and running distances. Based on the characteristic of zero velocity when the foot contacts the ground, the authors removed the drift error in the acceleration and then double-integrated the data to estimate the distance.

Table 1 summarizes recent results of walking distance estimation with different methods of attaching the sensors to the body. The body and leg attaching approaches provide high estimation results. The accuracy of walking distance measurement using the approach of attaching the sensors to the shoes is still low [15]. The estimated results are significantly improved in a recent work [16] with certain conditions.

These methods typically designed inertial sensor modules and then mounted them on the waist, thigh and/or ankle to collect biaxial/triaxial acceleration data. This approach has inherent disadvantages in real-life usage. One of the disadvantages is a serious inconvenience for users who must continuously wear these devices on their bodies during the experiment and data collection period.

Another approach of utilizing sensors to analyze gait is attaching the sensors to the insole of a shoe [20,21,22,23,24,25]. The advantage of this approach is that shoes are ubiquitous and typically worn on a continuous basis by humans. Therefore, attaching a sensor to the shoe’s insole does not cause inconvenience for the users, even over a long period. Moreover, installing measurement sensors in the insole of shoes can be an efficient method to track humans by walking distance measurement.

In this paper, we propose a novel method of walking distance estimation based on a precise stride counting and phase determination using an insole sensory module that consists of a triaxial accelerometer and eight pressure sensors. We incorporate pressure and acceleration information of healthy walkers to determine the swing and stance phases of their gaits. Evaluating experiments, we obtain the maximum error of the swing phase determination with respect to time being 0.08 s and 0.06 s for starting and stopping points of swing phases, respectively. The gait analysis results are then used to count the number of strides of walkers and estimate the walking distance. We apply a double integral to acceleration data to calculate the vertical displacement and the distance which the foot traces in the air. Based on this information, we estimate the walking distance in a straight line. The experimental results, which are conducted on various heights and ages of walkers, demonstrate the validity and accuracy of the measurement method. Specifically, we obtain error rates of 4.8% and 3.1% for experiments on a 16 m and 89 m walking distances, respectively.

The remainder of this paper is organized as follows. Section 2 presents the measurement system and estimation method based on acceleration and pressure data. A gait phase analysis is also presented to explain the walking distance measurement method. In Section 2, we also describe the process of determining the swing phase and the method of estimating the walking distance of the subjects. Section 3 shows the experimental results of the proposed method. Some important points are discussed in Section 4. Finally, conclusions are provided in Section 5.

## 2. Stride Counting and Walking Distance Estimation

In this section, we present the method to count strides and estimate walking distance based on gait cycle analysis. We explain the fundamental phases of the gait cycle and the application of gait analysis to the walking distance calculation. We also describe the insole module that is used to collect acceleration and pressure data of walking. In our proposed method, the pressure data are used to determine the starting and stopping points of the swing phases during the walking motion. This process also provides information for efficiently counting the number of strides of a walker. Then, we extract the acceleration data on the swing phases to calculate the walking distance. The walking distance is estimated based on the tracing distance of the shoe in the air and the vertical displacement of the shoe.

### 2.1. Gait Phase Analysis

**Figure 1:**
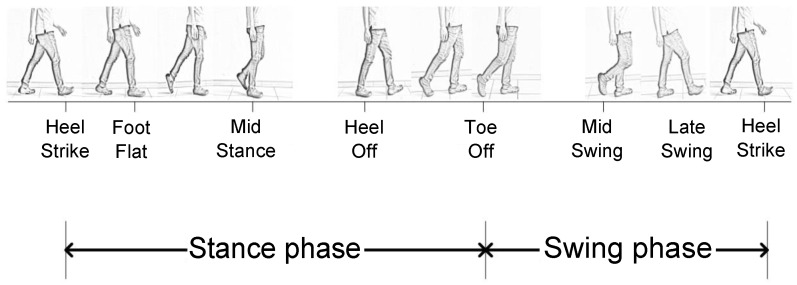
Analyzing phases of the gait cycle.

Typically, a gait cycle can be divided into seven phases [26,27,28], *i.e.*, heel strike, foot flat, mid stance, heel off, toe off, mid swing and late swing. The phases of a gait cycle are depicted in Figure 1. The heel strike indicates the period when the foot begins to contact the ground. The heel strike is the first phase of the gait cycle. The foot flat is the period where the body absorbs the impact of the foot by rolling in pronation. The mid stance phase begins when the other foot is lifted and continues until the body weight is pressed onto the forefoot. The heel off is the period where the heel begins to leave the ground. The toe off phase refers to the phase when the toe leaves the ground. In the toe off phase, the hip becomes less extended. In the mid swing, the adductors are contracted and the ankle is dorsiflexed by contraction of the tibialis anterior muscle. The late swing phase declares a locked extension of the knee and a neutral position of the ankle. A gait cycle ends at a new heel strike phase.

Using the right foot as a reference to apply the distance measurement, we categorize the gait cycle into two phases, *i.e.*, the stance phase and swing phase. The stance phase includes heel strike, foot flat, mid stance and heel off phases, whereas, the swing phase includes toe off, mid swing and late swing. The stance phase represents the time interval where the right foot is flat on the ground. The stance phase begins from the heel strike phase and ends at the toe off phase. After the stance phase ends, the swing phase begins. The swing phase refers to the period of time where the right foot is moving in the air and does not contact the ground. It should be noted that the movement distance is primarily determined by the swing phase of the gait cycle; thus, in the proposed approach, we identify the swing phase and then calculate the distance from the acceleration information collected in this phase. Specifically, we utilize a hardware module integrated into the shoe to collect the pressure and acceleration data of walkers’ gaits.

### 2.2. Insole Sensor-Based Estimation System

**Figure 2:**
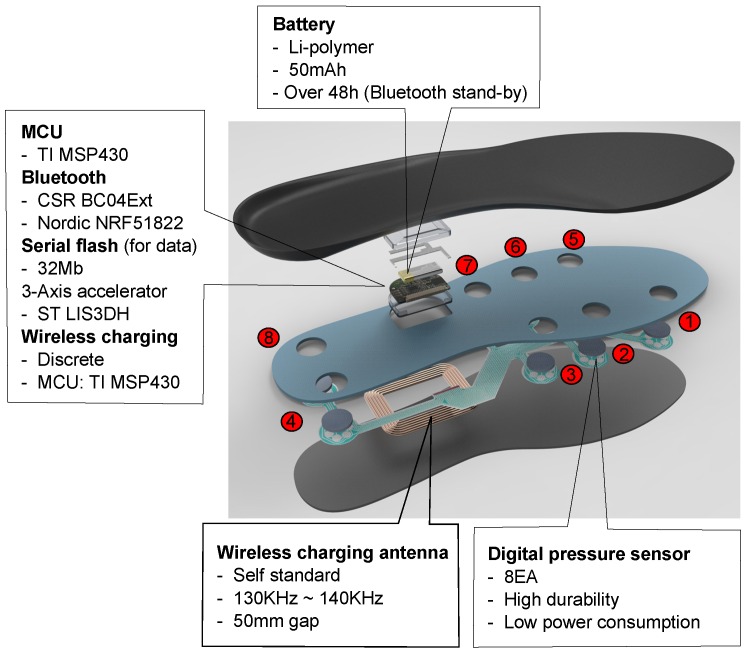
Design of the insole sensor module.

In this study, we use the insole sensor module, which was designed and developed by the 3L Labs Co., Ltd. (Seoul, Korea), to collect the data from the gaits. The insole sensor module is commercially called the “Footlogger”. The insole sensor module includes a triaxial accelerometer, eight pressure sensors attached to the insole of the shoe, and an microcontroller (MCU) kit supporting Bluetooth connection to transmit the movement information. Figure 2 illustrates the developed insole sensor module. In the experiments, the insole sensor module is connected to a smartphone to record the pressure and acceleration data from walkers. The sampling frequency of the insole sensor module is set to 50 Hz. Commonly, these sensors are sampled at frequency range of 20 Hz to 200 Hz [29]. In posture and activity classification, a low sampling rate of accelerometer and pressure sensor possibly produces excellent recognition accuracy [29,30]. In [30], pressure and acceleration data were sampled at 25 Hz by a 12-bit analog-to-digital converter to accurately identify sitting, standing and walking postures. In [29], authors stated that reduction of sampling frequency of accelerometer and pressure sensor from 25 Hz to 1 Hz does not create significant lost of accuracy (98% to 93%). In estimation applications, Aminian *et al.* [31] record body acceleration at a sampled rate of 40 Hz to estimate walking speed. The experimental results showed that the maximum of speed-predicted error is 16%. In [32], authors used the force transducers and inertial sensor in a shoe with the sampling rate of 50 Hz to estimate stride length. The root mean square (RMS) difference between the obtained stride length and the referenced stride length being 34.1 ± 2.7 mm. Based on the results of the related research, we believe that the sampling frequency of 50 Hz for accelerometer and pressure sensor in the system is possible to estimate walking distance.

To separate the gravity component from the raw acceleration signal, we first isolate the force of gravity with a low-pass filter. Then, we apply a high-pass filter to isolate the linear acceleration. The algorithm of extracting linear acceleration is as follows:

$$\begin{array}{ccc} g_{x}\left(t\right) & = & \alpha .g_{x}\left(t-1\right)+\left(1-\alpha \right)\cdot a_{x}^{r}\left(t\right),\\ g_{y}\left(t\right) & = & \alpha .g_{y}\left(t-1\right)+\left(1-\alpha \right)\cdot a_{y}^{r}\left(t\right),\\ g_{z}\left(t\right) & = & \alpha .g_{z}\left(t-1\right)+\left(1-\alpha \right)\cdot a_{z}^{r}\left(t\right),\\ a_{x}\left(t\right) & = & a_{x}^{r}\left(t\right)-g_{x}\left(t\right),\\ a_{y}\left(t\right) & = & a_{y}^{r}\left(t\right)-g_{y}\left(t\right),\\ a_{z}\left(t\right) & = & a_{z}^{r}\left(t\right)-g_{z}\left(t\right) \end{array}$$

where $\left\{g_{x},g_{y},g_{z}\right\},\left\{a_{x}^{r},a_{y}^{r},a_{z}^{r}\right\}$ and $\left\{a_{x},a_{y},a_{z}\right\}$ are gravity, raw acceleration and linear acceleration on *X*-,*Y*- and *Z*-axes, respectively. Moreover, we select α=0.8 for the low-pass filter.

**Figure 3:**
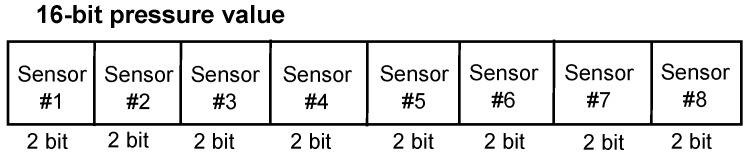
Fusing sensors’ values.

The inertial triaxial accelerometer sensor is placed in the center of the insole. The pressure sensors are placed at the rear of the shoe. Each pressure sensor contains a two-bit value representing the pressing level on the sensor. To effectively detect the gait phases of walkers, we fuse the data from these eight pressure sensors into a pressure value. The incorporate pressure value on the shoe is determined by a 16-bit value created from these eight two-bit values of the sensors, where the two bits of the first sensor are the most significant bits and the two bits of the eighth sensor are the least significant bits, as shown in Figure 3. This fusing method is designed to emphasize the importance of pressure on the first metatarsal area because this area is larger pressed compared with the fifth metatarsal area in straight walking.

**Figure 4:**
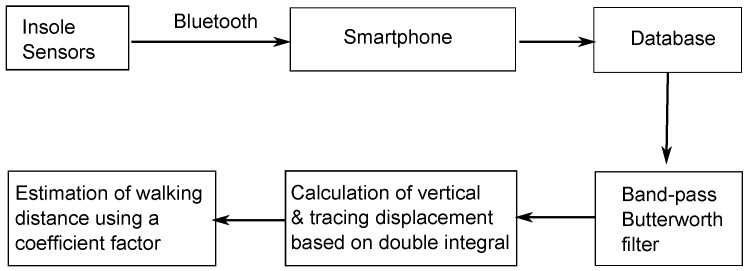
Diagram of data processing in the estimation system.

Using this insole sensor and a smartphone, we created our portable and concise system for estimating walking distance. We attached the sensor module, which includes sensors and batteries, into an insole and then placed it inside a shoe. The insole used in the system is the off-the-shelf insole, whereas, the experimental shoe is user’s sneaker. This design is to make users feel comfortable during their experiments, and thus ensure that their gaits are not affected. Indeed, we did not receive any complaints from experimental subjects about the discomfort of the insole during experimentation. Thus, we can provisionally conclude that the insole sensor module is unlikely to affect users’ gaits. The insole sensor is connected to the smartphone through Bluetooth to continuously transfer collected data. Figure 4 shows the flow of data in the estimation system. We use the sensor module in the insole of the shoe for the right foot and require walkers to wear this shoe to collect data during the walking period. The collected data are sent to a smartphone in the pocket of the walkers for storage and processing through the Bluetooth connection. We remove noise in the data received from the foot-worn sensors using a band-pass Butterworth filter with $f_{c1}$ = 5 Hz, $f_{c2}$ = 10 Hz. Finally, we apply our calculation method on these data to estimate the walking distance.

### 2.3. Stride Determination

**Figure 5:**
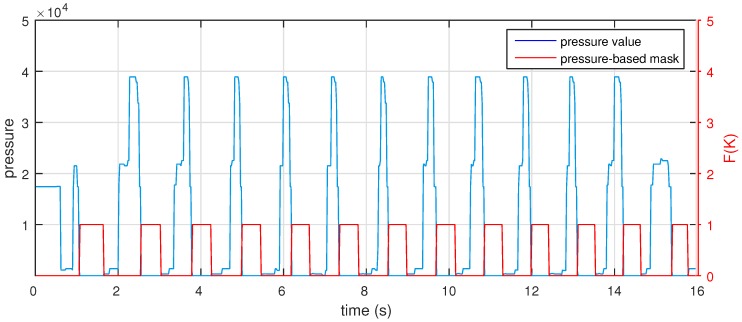
Swing phase determination based on pressure information.

It is clear that the number of walking strides equals the number of swing phases, which is the period immediately after a user pushes off his foot, releasing it from the ground until the foot again contacts the ground. Therefore, to detect swing phases, and thus possibly count the number of walking strides, we analyze the pressure under the walker’s shoes. We basically utilize the data received from pressure sensors to determine the time interval of the swing phase. When a walker releases his foot from the ground, the pressure value of the sensor under his foot will be virtually zero, whereas when the foot contacts the ground, the pressure value is not zero. Based on this fact, we create an on/off filter to detect swing phases of walking. If all pressure sensors point to “not press” state, the foot is defined as being in swing phase. However, we experimentally observed that Sensor 8 sometimes provides a level of “slightly pressed”, corresponding to the $01_{2}$ value in the binary system, when users transit from late swing phase to heel strike phase and are still in the late swing phase. Therefore, to remove this effect, we set a threshold corresponding to the wrong case of Sensor 8, *i.e.*, threshold = 1. This threshold-based method works because the swing phase is only identified when all sensors are in “off” state. This means that all sensors should point to the “no press” state. Applying these analyses, we create a simple filter to detect the period of the swing phase in human walking:

(1) $$F\left(k\right)=\left\{\begin{array}{cc} 0 & \mathrm{if}\;p(k)<threshold\\ 1 & \mathrm{if}\;p(k)\geq threshold \end{array}\right.$$

where *p* is the pressure data received from the foot logger at the *k* sampling index. Figure 5 illustrates the scenario of a pressure value received from walking in a 16 s walking period. The blue part represents the pressure data measured by the pressure sensor integrated under the users’ shoes. The red part shows the result F(k) of the on/off filter presented in Equation (1). In other words, F(k)=1 means the insole is in swing phase at the *k* moment. Based on this pressure-based swing phase filter, we can determine the starting and stopping moments of swing phases as well as movement.

### 2.4. Human Walking Distance Estimation

**Figure 6:**
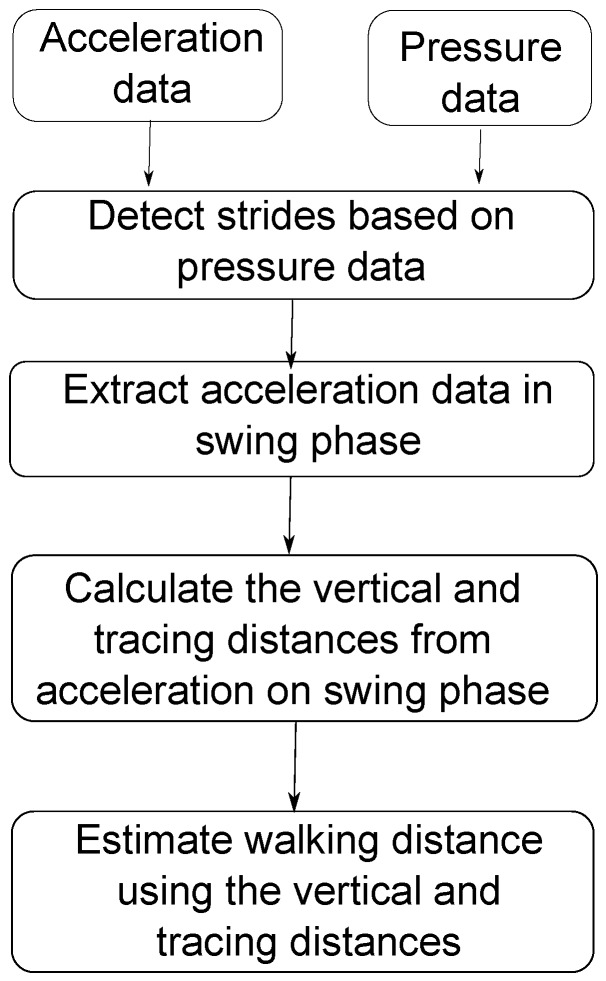
Proposed calculation algorithm.

Based on the estimation of the swing phases, a method based on double-integration of acceleration data is used to calculate the movement distance in the swing phase. We use a double integral to compute the vertical movement distance and the tracing distance of the foot in the air. Using these two values, we estimate the walking distance. The proposed calculation algorithm is generalized in Figure 6.

#### 2.4.1. Swing-Phase Acceleration Extraction

The pressure-based swing phase filter is used to extract the corresponding acceleration data of the walking cycle. A simple convolution is applied to extract feasible data from the acceleration data:

(2) $$\begin{array}{cc} {\hat{a}}_{x}\left(k\right) & =F\left(k\right)*a_{x}\left(k\right)\\ {\hat{a}}_{y}\left(k\right) & =F\left(k\right)*a_{y}\left(k\right)\\ {\hat{a}}_{z}\left(k\right) & =F\left(k\right)*a_{z}\left(k\right) \end{array}$$

**Figure 7:**
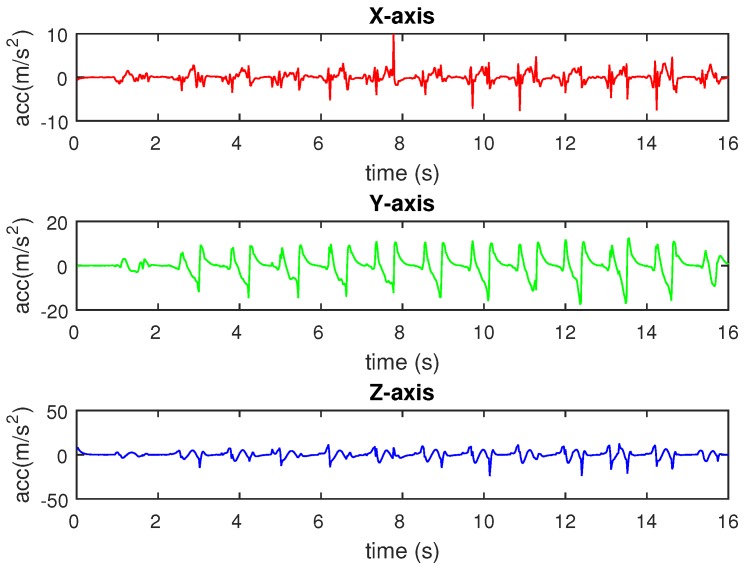
Original acceleration data.

**Figure 8:**
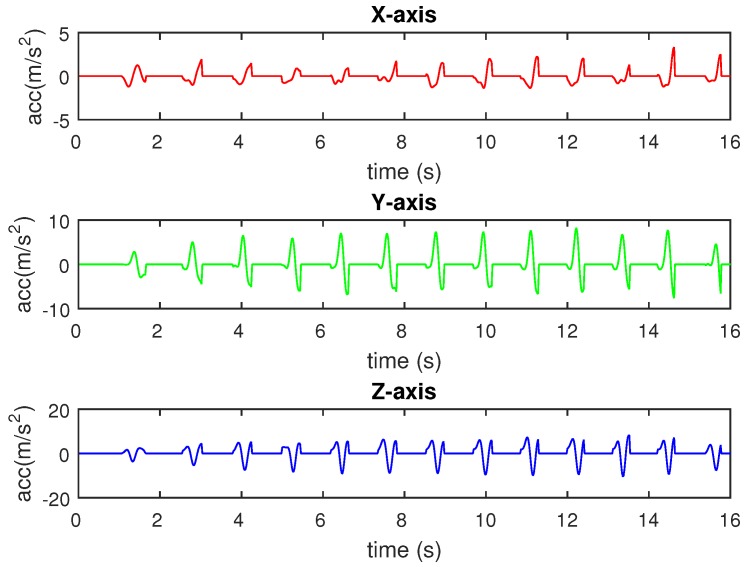
Extracting filtered acceleration data using pressure-based filter.

In Equation (2), ${\hat{a}}_{x}\left(k\right)$, ${\hat{a}}_{y}\left(k\right)$, and ${\hat{a}}_{z}\left(k\right)$ are the swing phase extracted acceleration on the *X*-, *Y*-, and *Z*-axes, respectively, whereas $a_{x}\left(k\right)$, $a_{y}\left(k\right)$, $a_{z}\left(k\right)$ are the original collected acceleration on the *X*-, *Y*-, and *Z*-axes, respectively, and F(k) is the value of the filter at the *k* sampling index. Figure 7 shows a sample of the raw triaxial acceleration measured by the sensing module. The results of filtering the measured acceleration are depicted in Figure 8. The filter allows acceleration data in swing phases to pass and removes acceleration components in stance phases on three axes.

Based on this filtering, we can determine the period of swing in the walking motion. Moreover, with this pressure-based filter, we can easily calculate the exact number of strides in the walking distance. After extracting acceleration in swing phases, we apply double integral-based calculation to estimate stride lengths. Therefore, the walking distance can be measured.

#### 2.4.2. Distance Estimation with Continuous Data

To determine the walking distance, we measure the tracing distance of the shoe, and then multiply the result with a correction coefficient to estimate the movement distance of a walker in a straight line. First, we obtain the acceleration data in the swing phase. Then, we double-integrate the obtained acceleration data to calculate the tracing and vertical displacements during the swing phase. The tracing displacement mentioned is the curved line on which the foot traces in the air, whereas, the vertical displacement indicates the movement in the *Z*-axis of the sensor frame. Based on these two values, we estimate the distance traveled in swing phase. Finally, we add the displacement caused by the foot length during walking into the result to obtain the estimated value. Basically, the distance is estimated from the acceleration data using the motion equation of the foot:

(3) $$d=K\cdot \frac{\int_{0}^{T}\left(\int_{0}^{T}a\left(t\right)\;dt\right)dt}{\int_{0}^{T}\left(\int_{0}^{T}a_{z}\left(t\right)\;dt\right)dt}+N\cdot L_{0}$$

where *T*, *N*, $L_{0}$ and *K* are the time period of walking, the stride number, the constant foot length and an experimental coefficient, respectively, whereas $a\left(t\right)=\sqrt{a_{x}^{2}\left(t\right)+a_{y}^{2}\left(t\right)+a_{z}^{2}\left(t\right)}$ is the acceleration obtained in the swing phase.

#### 2.4.3. Distance Estimation with Sampling Data

Applying the estimation method mentioned in Section 2.4.2, we compute the acceleration magnitude:

(4) $$a\left[k\right]=\sqrt{a_{x}^{2}\left[k\right]+a_{y}^{2}\left[k\right]+a_{z}^{2}\left[k\right]}$$

Then, the distances traveled by the foot in the air and in the vertical direction of each stride are calculated as follows:

(5) $$\begin{array}{ccc} v_{MAG} & = & \sum_{k=1}^{N}a[k]\cdot \Delta t\\ d_{MAG} & = & \sum_{k=1}^{N}v_{MAG}\left[k\right]\cdot \Delta t \end{array}$$

(6) $$\begin{array}{ccc} v_{z} & = & \sum_{k=1}^{N}a_{z}\left[k\right]\cdot \Delta t\\ d_{z} & = & \sum_{k=1}^{N}v_{z}\left[k\right]\cdot \Delta t \end{array}$$

where *N* and Δt are the number of sampling times and the time period of sampling, respectively.

Applying this result, the walking distance of the walker can be estimated in online mode using the experimental smartphone. The smartphone periodically receives sensors’ data from the insole sensor module under the user’s shoe to estimate the traveled distance. Specifically, the traveled distance at the *n* stride index, d[n], can be calculated from the estimated distance at the previous stride index, d[n−1], and acceleration data in the *n* stride index by:

(7) $$d\left[n\right]=d\left[n-1\right]+K\cdot \frac{d_{MAG}\left[n\right]}{d_{z}\left[n\right]}+L_{0}$$

where $d_{MAG}\left[n\right]$ and $d_{z}\left[n\right]$ are the distance obtained using magnitude and vertical acceleration in the time interval of the *n* stride index, respectively. The coefficient *K* is tuned to a value so that the mean value of estimated distances in each training set is equal to the reference distance.

### 2.5. Descriptive Analysis and Requirements

This section descriptively analyzes the experimental requirements and explains related features of subjects who joined the experiment. The experiments were conducted on totally seven healthy subjects consisting of two female and five male walkers, aged from 22 to 28 years old, with height varying from 160 cm to 185 cm. Table 2 describes the baseline of individuals including personal information of each person, *i.e.*, height, age and sex. The average foot length is chosen as $L_{0}$ = 0.26 m to estimate the distance traveled. In Table 2, we also mark the specific distance each subject walks to create the walking databases. All seven subjects participated the 16 m distance walking, whereas three of them participated in the 89 m distance walking experiment. The experiments were conducted in an indoor environment, *i.e.*, a building of a university. Initially, subjects stood with both feet flat on the ground. Then, we asked them to step on the distances leading with the right foot. The subjects should lift up their right foot first from the initial state. During walking, subjects are required to lift their feet from the ground in each swing phase. The subjects are discouraged to drag their feet on the ground in swing phases, which can affect the pressure-based gait analysis.

**Figure 9:**
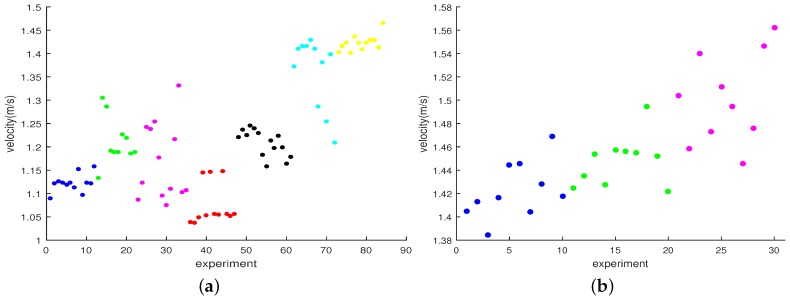
Gait velocities of subjects in experiments. (**a**) short distance database; (**b**) long distance database.

The subjects were required to walk normally in the straight distances with the insole sensor module installed in their right shoes and an Android smartphone to collect the acceleration and pressure information from the insole sensors. The smartphone connected with the insole sensor using Bluetooth and seamlessly received the data from the insole sensor module. We collected 84 samples of data of walking on the 16 m distance from the seven subjects to create the short distance walking database. Similarly, the long distance database was created based on 30 walking samples of three subjects on the 89 m distance. This group of three subjects is a part of the group of seven subjects who conducted experiments of short distance walking. The specific participation of each subject in experiments is shown in the last two columns of Table 2. The walking speed measured from all subjects varied in the range of 1 m/s to 1.6 m/s, specifically, v=1.216±0.127 m/s. Figure 9 shows the distribution of subjects’ average speeds in the experiments of walking on 16 m and 89 m distances. Different subjects are represented by different colors in experiments. The same color in the two subfigures illustrates the same subject in two databases.

## 3. Experimental Results

This section describes the preliminary experimental results that demonstrate the accuracy of the proposed calculation method using the insole sensor module. These experiments were conducted in a preliminary trial where walkers were required to walk normally for a specified distance in a straight line. Movement data that were collected using the sensor module were utilized to create a database for evaluating the accuracy of the proposed method.

### 3.1. Stride Counting

**Figure 10:**
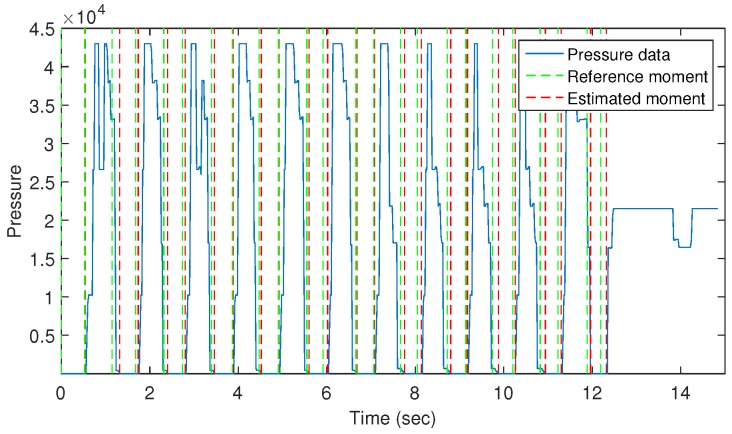
Determination of swing phases on a walking sample.

Using the pressure data to analyze the gait cycle, we can accurately estimate swing phases’ moment, and hence, an extremely precise result for stride counting can be calculated. To evaluate, we used a camera to record all subject movement during the walking periods. Specifically, we handle the camera and follow walkers to record their walking data. Using the recorded video, we manually marked the starting and stopping moments of swing phases to identify the reference information of swing moment. Compared with the estimated moment received from the swing phase determination, we obtained the maximum difference of 0.08 s and 0.06 s for the starting and stopping points, respectively. Figure 10 shows the difference between the estimated and reference moments of swing phases in a walking sample on the 16 m distance. Based on the swing phase determination, we counted the number of strides and compared this with the result received from the pressure analysis-based method to validate its accuracy.

Applying the swing phase detection into the stride counting application, we obtained results with an accuracy of 100%. These results confirm that by applying the pressure based filter, stride counting can be precisely calculated. It is clear that, with the support of hardware, acceleration-based gait analysis can be easily accomplished. In other words, analyzing pressure under shoes facilitates determining the stance phase of walking which is a difficulty for acceleration analysis-based methods.

### 3.2. Walking Distance Estimation

To statistically evaluate the accuracy of selecting the experimental coefficient *K* for the proposed method, we divided the database into the training set and test set using Leave-One-Out cross validation. Specifically, we divide the short distance database into a training set of 83 samples and a test set of one sample to evaluate the proposed. We alternatively repeat this division 84 times so that every sample is used as a test set one time. Similarly, we split 30 samples of the long distance database into a training set of 29 samples and a test set of one sample. This split is alternatively conducted 30 times; hence, each sample is used in training 29 times and in testing one time. Using the training set, we acquire the required coefficient *K* for each sample. Then, we obtain the average value of all these coefficients to select the final experiment coefficient. Applying this selected experiment coefficient to the test set, we calculate the walking distance and measurement error. The measurement error on N samples were calculated as follows:

(8) $$e=\frac{1}{N}\sum_{i=1}^{N}\frac{\left|d^{\left(m\right)}-d^{\left(r\right)}\right|}{d^{\left(r\right)}}$$

where $d^{\left(m\right)}$ and $d^{\left(r\right)}$ are the measurement and reference distances, respectively.

To evaluate the accuracy of the proposed algorithm, we tested the algorithm on two databases, *i.e.*, the short-distance (16 m) and long-distance (89 m) databases. Table 3 shows the estimation results on the short- and long-distance databases. We determined the mean, median, min, max and standard deviation (SD) values of the estimation, then calculated the average relative error for each subject to depict estimation distribution on each subject. We also calculated these values for all recorded samples of the databases to test the accuracy of the proposed method. In summary, for the 16 m-distance database, the proposed method provided an estimation result of 16 m ± 1 m with the accuracy rate of 95.2%. On the 89 m-distance database, we obtained the estimation result of 89 m ± 3 m with the accuracy rate of 96.9%.

In Table 4, we compare the estimation results of the proposed method with other reference methods. The method proposed by Alvarez [15] used a double integral of acceleration on the movement direction of foot sensors and drift removal to measure the walking distance. The method [19] utilized double integral with zero velocity update (ZUPT) to estimate walking distance based on acceleration data collected from foot sensors. The ZUPT technique was performed by extracting acceleration between two neighboring zero velocity windows, then shifted velocity to remove drift error. The experimental results clearly show that the proposed method outperforms referenced estimation methods in terms of estimation error and deviation.

## 4. Discussion

In our proposed method, we use a tuned coefficient *K* to obtain the final estimation results from calculated displacements. This coefficient is not only a unit converter, but also a correction for the estimation. Indeed, this tuned coefficient shifts the mean value of calculation results to the reference value. It can be said that the horizontal displacement and coefficient *K* play a role of projecting the tracing distance on each strides to the horizontal direction of the global frame.

The proposed method uses the merging pressure data to detect stance and swing phase. It should be noted that this pressure data is not the physical pressure obtained at the insole. Instead, the pressure data are the merging of pressure levels from eight pressure sensors. This merging ensures eight pressure levels separate in the final result. Furthermore, this merging technique facilitates data communication between the insole sensor module and smartphone. Instead of separately transferring eight pressure values, we only transfer one merging pressure value at each sampling moment. Currently, we just classify swing and stance phases; thus, only the on/off information is used. A lower value of merging pressure that is created as an 8-bit value with each bit for each sensor’s state can provide the same result. However, spending implementation time in smartphone for creating this lower value is unnecessary because the two-phase classification can be done directly from the 16-bit value. Moreover, for further gait analysis, such as detect heel-strike phase, this differentiation of level is necessary. In future works, we plan to use these pressure data to classify gait phases defined in Section 2.1 to apply in other related applications such as, fall detection or rehabilitation.

Another important point to discuss is that the gait analysis presented in Section 2.1 is only for a walking model. When a subject begins walking from standing state, his/her first gait is not corresponding to this gait analysis. The first gait starts from mid stance phases and ends at the heel strike phase. Its stance phase includes mid stance and heel off phases, but excludes heel strike and foot flat phases. In fact, the first gait is a step, not a stride. However, in our experimental requirement, walkers initially stand with both feet on the ground, and then step on the distances leading with the right foot. Moreover, our estimation method is created based on results of a double integral of acceleration data on swing phases. Using the presented pressure-based filter, we are still able to determine the swing phase of this first stride. Then, we can extract acceleration data and apply the proposed method to estimate the first stride length. Therefore, we consider the first step as a short stride. We assume the first gait is a short stride including stance and swing phases. With this assumption, the proposed method can be smoothly applied for walking distance estimation. The assumption is reasonable because the distance traveled is basically determined by the swing phase. The distance traveled in the swing phase is estimated based on tracing and vertical displacements. Technically, these displacements are dependent on acceleration data and walking period. The first stride period is shorter than other strides’ periods; thus, we believe that this assumption is practically applicable.

In this paper, we used the estimation system to detect stance and swing phases, and then estimate walking distance on healthy subjects. The experiments were conducted with a controlled initial state and a requirement during walking. These are subjects that should initially stand with both feet on the ground and walk without dragging their feet on the ground. If applying the method on subjects who suffer from gait disorders, the accuracy of the estimation can be reduced. A pathological gait can affect the proposed method by the following points.

- Patients drag their foot on the ground;
- Gait disorder can cause misunderstanding in stride determination;
- Correlations between the tracing and horizontal displacements with the walking distance can be various.

To solve these problems, the pressure-based gait analysis should be improved. The band-pass Butterworth filter should be also edited. We think that, to better estimate walking distance for both healthy and pathological gaits, the estimation method should be combined with a classification of gait. Before applying the walking distance estimation, a gait classifier should be used to choose an appropriate model for estimation.

## 5. Conclusions

In this paper, using a sensor module consisting of an inertial triaxial accelerometer and eight pressure sensors, we proposed a novel method of walking distance estimation based on an accurate phase determination and precise stride counting. The sensor module was installed in the insole of the shoe to collect human walking information. The information was filtered and processed on the smartphone to remove high frequency noise. Using the pressure data, we accurately estimate the starting and stopping moments of swing phases and precisely count the number of walking strides of each subject in the experiments. Double-integrating the acceleration data in the swing phase provided the foot’s tracing displacement in the air and the vertical displacement of the foot in the sensor frame. Based on the tracing in the air and vertical displacements of the foot, we estimate the anterior walking distance of subjects on a straight line. The experimental results confirmed that the proposed method performed accurately with a mean walking distance estimation error of 4.8% and 3.1% for 16 m and 89 m walking distance.

Currently, the proposed method can only be applied on estimating walking distance of normal walking on level ground. In a future work, we will extend the proposed method with various walking patterns, such as stair climbing, jogging, and running. We also plan to estimate the walking directions with an integrated gyroscope sensor, and create a PDR system using the insole sensor integrated shoe. As stated previously, we plan to use the pressure data to classify gait phases defined in Section 2.1 and expand the research into other areas such as, abnormal gait recognition, fall detection or rehabilitation.

## Figures and Tables

**Table 1:** Comparison of walking distance estimation approaches.

Approach	Author	Assumption	Method	Results
Body attaching	Shin *et al.* [17]	- Pedestrians walk or run - Attaching accelerometers to the body	- Using biaxial accelerometer and gyroscope sensors - Counting the number of steps - Estimating the step length as a linear combination of walking frequency and acceleration variance.	- Accuracy of step length estimation for walking cases is 95%, 96% and 96% for slow, normal, and fast walking, respectively - Step length estimation provides an accuracy of 96% for running case
	Shih *et al.* [12]	- Users walk normally in a straight line with average distance of 664.5 cm. - Attaching smartphone on the waist - Placing smartphone on the chest pocket	- Using one triaxial accelerometer and one gyroscope sensor from a smartphone - Using double integral of vertical acceleration to estimate stride length	- Accuracy of distance estimation based on attaching the smartphone on the waist is 97.35%. - Placing smartphone on the chest pocket provides a 96.14% accuracy rate of distance estimation
Ankle attaching	Wang *et al.* [9]	- Users walk along the outside of a sports area that is 559 m long - Attaching triaxial accelerometer on users’s ankles	- Using a triaxial acceleration data to analyze gait and estimate the step velocity - Estimating step length as a linear regression model of step frequency and step velocity	- Accuracy of walking distance estimation is 96.42%
Leg attaching	Bennett *et al.* [18]	- Subjects walk in a straight line with average distance of 3.55 m - Placing sensors on the thigh and shin of the right leg	- Modeling human leg as a two-link revolute robot, then using Extended Kalman Filter (EKF) to estimate the displacement in a straight line	- EKF distance estimation had an average error of 2%
Shoe attaching	Alvarez *et al.* [15]	- Subjects walk in a 10 m straight distance - Attaching a sensor module in the front of the users’s shoes	- Using a biaxial accelerometer and a gyroscope sensor - Double integrating the horizontal acceleration in the swing phase to estimate the walking distance.	- Mean estimation error rate is 10% with a single sensor module attached on one foot - Result is improved to 7% when mounting a sensor module on each foot
	Wang *et al.* [19]	- Three subjects perform two sets of 40 m level walking, 10-step stair ascending and 10-step stair descending - Mounting a triaxial accelerometer, a gyroscope and orientation sensors to shoes	- Using double integral of acceleration to estimate walking distance - Using zero velocity update (ZUPT) to reset velocity when a foot becomes stationary	- Absolute error of (3.08±1.77)% in distance estimation
	Meng *et al.* [16]	- Subjects walk in a straight line for 10 m - In long distance experiment, subjects walk for a distance of approximately 132 m - Attaching an inertial/ magnetic measurement unit in the front of the users’ shoes	- Using a module containing a triaxial accelerometer, a triaxial gyroscope sensor and a triaxial magnetometer - Creating a zero velocity update method based on the stride information to further correct the acceleration - An adaptive Kalman Filter is used to estimate the position	- Position error is 0.44 m ± 0.2 m for short distance (4.4%). - In long distance experiment, the position error is 4.31 m ± 1.77 m (3.6%)

**Table 2:** Baseline of individuals.

Subject	Height	Age	Sex	Short Distance	Long Distance
#1	163 cm	23	Male	✓	✓
#2	165 cm	23	Male	✓	✓
#3	168 cm	28	Male	✓	✓
#4	175 cm	24	Male	✓	
#5	185 cm	26	Male	✓	
#6	160 cm	22	Female	✓	
#7	162 cm	26	Female	✓	

**Table 3:** Estimated values using the proposed method.

Distance	Subject	Mean (m)	Median (m)	Min (m)	Max (m)	SD (m)	Error
short (16 m)	#1	16.4	16.3	16.1	17.1	0.3	2.2%
	#2	15.4	15.4	14.4	16.6	0.6	4.5%
	#3	17.1	17.1	16.8	17.3	0.4	6.7%
	#4	15.7	15.4	13.9	17.3	1.4	8.0%
	#5	17.0	17.1	15.5	18.8	1.0	7.1%
	#6	15.3	15.3	14.9	15.7	0.2	4.5%
	#7	16.1	15.7	15.2	19.2	1.0	3.9%
	All	16.0	15.9	13.5	19.7	1.0	4.8%
long (89 m)	#1	88.4	88.4	87.4	89.4	0.7	0.8%
	#2	93.0	92.9	91.1	94.6	1.1	4.5%
	#3	85.6	85.7	83.3	87.3	1.4	3.9%
	All	89.0	88.1	83.8	94.5	3.2	3.1%

**Table 4:** Comparison with other methods.

Distance	Criterion	[15]	[19]	Proposed Method
short (16 m)	Error	17.5%	9.9%	4.8%
	SD	20.4%	13.1%	6.6%
long (89 m)	Error	10.4%	7.2%	3.1%
	SD	12.3%	8.8%	3.6%
